# Microevolution of Highly Pathogenic Avian Influenza A(H5N1) Viruses Isolated from Humans, Egypt, 2007–2011

**DOI:** 10.3201/eid1901.121080

**Published:** 2013-01

**Authors:** Mary Younan, Mee Kian Poh, Emad Elassal, Todd Davis, Pierre Rivailler, Amanda L. Balish, Natosha Simpson, Joyce Jones, Varough Deyde, Rosette Loughlin, Ije Perry, Larisa Gubareva, Maha A. ElBadry, Shaun Truelove, Anne M. Gaynor, Emad Mohareb, Magdy Amin, Claire Cornelius, Guillermo Pimentel, Kenneth Earhart, Amel Naguib, Ahmed S. Abdelghani, Samir Refaey, Alexander I. Klimov, Ruben O. Donis, Amr Kandeel

**Affiliations:** Author affiliations: US Naval Medical Research Unit No.3, Cairo, Egypt (M. Younan, E. Elassal, M.A. ElBadry, S. Truelove, A.M. Gaynor, E. Mohareb, C. Cornelius, G. Pimentel, K. Earhart);; Centers for Disease Control and Prevention, Atlanta, Georgia, USA (M.K. Poh, T. Davis, P. Rivailler, A.L. Balish, N. Simpson, J. Jones, V. Deyde, R. Loughlin, I. Perry, L. Gubareva, A.I. Klimov, R.O. Donis);; Cairo University, Cairo (M. Amin);; Ministry of Health and Population, Cairo (A. Naguib, A.S. Abdelghani, S. Refaey, A. Kandeel)

**Keywords:** H5N1 avian influenza virus, evolution, highly pathogenic, Egypt, poultry, genomics, influenza, viruses, highly pathogenic avian influenza A(H5N1), subtype H5N1, avian influenza, zoonoses

## Abstract

We analyzed highly pathogenic avian influenza A(H5N1) viruses isolated from humans infected in Egypt during 2007–2011. All analyzed viruses evolved from the lineage of subtype H5N1 viruses introduced into Egypt in 2006; we found minimal evidence of reassortment and no exotic introductions. The hemagglutinin genes of the viruses from 2011 formed a monophyletic group within clade 2.2.1 that also included human viruses from 2009 and 2010 and contemporary viruses from poultry; this finding is consistent with zoonotic transmission. Although molecular markers suggestive of decreased susceptibility to antiviral drugs were detected sporadically in the neuraminidase and matrix 2 proteins, functional neuraminidase inhibition assays did not identify resistant viruses. No other mutations suggesting a change in the threat to public health were detected in the viral proteomes. However, a comparison of representative subtype H5N1 viruses from 2011 with older subtype H5N1 viruses from Egypt revealed substantial antigenic drift.

Outbreaks of highly pathogenic avian influenza (HPAI) A(H5N1) virus infection among poultry in parts of Africa, the Middle East, and Asia have caused sporadic human infections with high case-fatality ratios and a few instances of possible human-to-human transmission (*1*). In Egypt, HPAI (H5N1) virus was first detected in poultry in February 2006, and in March 2006, the first human infection was detected (*2*). Surveillance in wild and domestic birds and phylogenetic analyses of viruses from the region indicated that subtype H5N1 virus was probably transported to Egypt by wild birds migrating from the Qinghai Lake region of the People’s Republic of China in the fall of 2005 (*3*). Analyses of the evolution of subtype H5N1 viruses in Egypt in the following years showed the exclusive circulation of clade 2.2 viruses in poultry, indicating no other subtype H5N1 viruses had been introduced (*4*). Subsequent genetic divergence of the clade 2.2 hemagglutinin (HA) genes in Egypt, however, resulted in a distinct phylogenetic group designated clade 2.2.1, which is enzootic in peridomestic poultry, and a more recently classified sister group known as clade 2.2.1.1, which is mostly found in commercial chicken flocks (*4*–*6*).

In 2011, a total of 39 human cases of subtype H5N1 infection and 15 related deaths were reported in Egypt; a total of 158 cases and 55 deaths were reported during 2006–2011, making Egypt the country with the second highest number of human cases after Indonesia (*7*). Increases in the annual number of human infections and accompanying decreases in case-fatality ratios in Egypt during 2009–2011, compared with those in 2006–2008, led to the hypothesis that the circulating viruses may have acquired distinct virologic properties (*8*). To address this issue, we studied the genetic and antigenic diversity of subtype H5N1 viruses isolated from humans in Egypt during 2007–2011. Analysis of 90 complete viral genomes was conducted to determine 1) the predominant genotype of subtype H5N1 viruses infecting humans and 2) other molecular changes that would suggest altered phenotypic properties, susceptibility to antiviral drugs, or the need for development of new candidate vaccine viruses.

## Materials and Methods

### Virus Isolation and Sequencing

We extracted RNA from clinical samples from patients with suspected HPAI infection by using the QIAmp Viral RNA Mini Kit (QIAGEN Inc., Valencia, CA, USA), according to the manufacturer’s protocol. We performed real-time reverse transcription PCR using the Centers for Disease Control and Prevention’s (CDC) primer-probe pairs for the detection of influenza A (matrix gene) or H5a and H5b (HA gene), as described (*9*). Positive results were confirmed at the Central Public Health Laboratory operated by the Egyptian Ministry of Health and Population and at the US Naval Medical Research Unit No. 3 in Cairo, Egypt. Viruses were isolated from PCR-positive clinical samples in embryonated chicken eggs. All procedures using live virus were conducted in biosafety level 3 facilities with enhancements recommended by the United States Department of Agriculture.

Overlapping amplicons of each gene segment were generated by reverse transcription PCR using subtype H5N1 virus–specific primers (primer sequences are available upon request). We then sequenced the amplicons by using the BigDye Terminator v3.1 Cycle Sequencing Kit (Applied Biosystems, Foster City, CA, USA). Sequences were assembled and edited by using Sequencher 4.9 (Gene Codes Corp., Ann Arbor, MI, USA).

### Phylogenetic Analysis and Molecular Characterization

**Figure 1:**
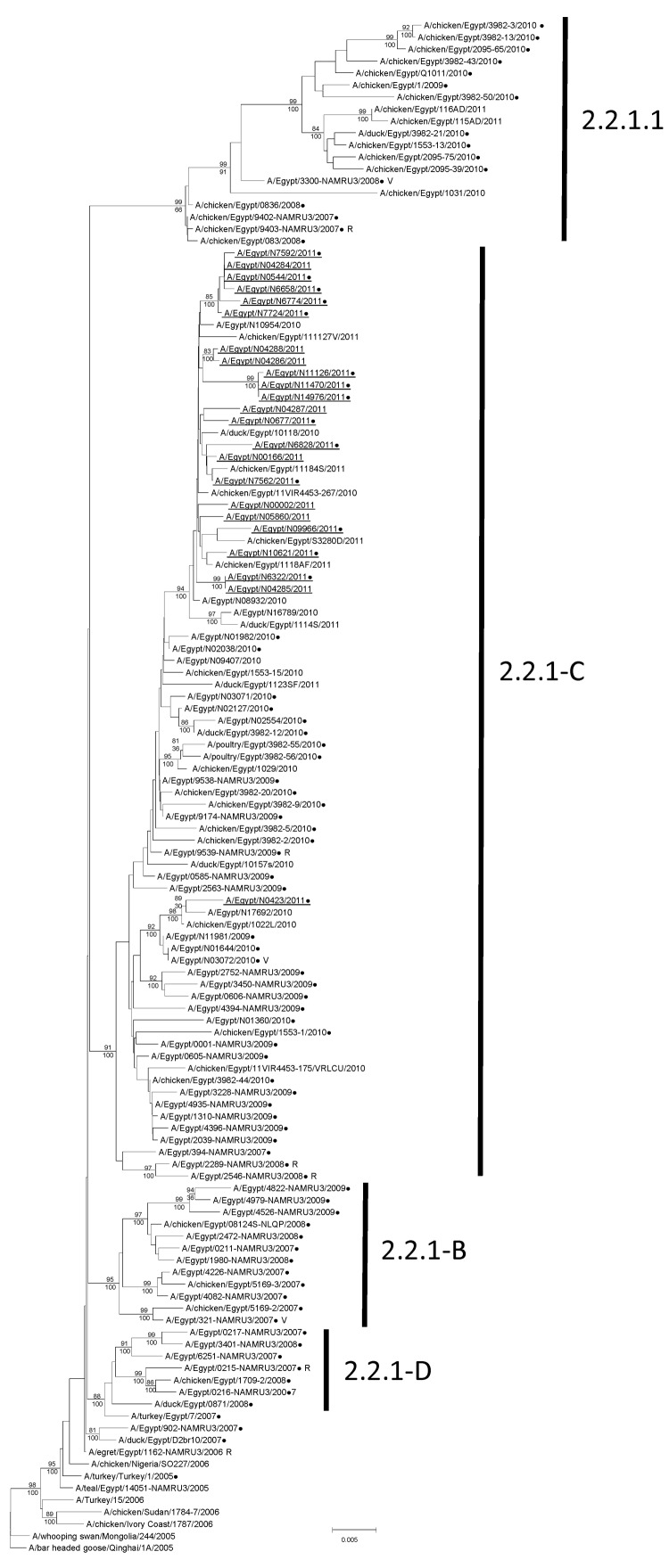
Phylogenetic tree of the influenza A(H5) virus hemagglutinin genes, clade 2.2.1, generated by neighbor-joining analysis. Subgroups of clade 2.2.1 are indicated on the right. Bootstrap values (>79) generated from 1,000 neighbor-joining replicates are shown above branches, and Bayesian posterior probabilities are shown below the branches at relevant nodes. Scale bar represents 0.002 nt substitutions per site. Viruses recommended by the World Health Organization for development of candidate pandemic vaccines are indicated with a V; viruses inoculated into ferrets to raise antiserum for hemagglutinin inhibition assays are indicated with an R. Underlined names denote 23 human viruses collected in 2011. Sequences used for full genome analysis in this study are annotated with a dot.

For analysis, we used a total of 59 subtype H5N1 viruses from human clinical specimens (sequenced for this study) and 31 subtype H5N1 viruses from birds (from public databases) collected in Egypt during 2007–2011 (Technical Appendix 1). In addition, we sequenced 13 HA genes directly from RNA extracted from clinical specimens. Nucleotide sequences were aligned in BioEdit (Ibis Biosciences, Carlsbad, CA, USA) using MUSCLE (*10*). We used full-length HA sequences for viruses collected during 2006–2011 to generate the clade 2.2.1 and 2.2.1.1 HA gene phylogeny; the sequences comprised 72 genes sequenced by CDC and US Naval Medical Research Unit No. 3 and 56 publicly available HA sequences (Figure 1). Sequences were aligned and trees were built with MEGA5 software (*11*), using the neighbor-joining method based on a maximum composite likelihood model. The reliability of the trees was estimated by bootstrap resampling analysis (1,000 replications).

**Figure 2:**
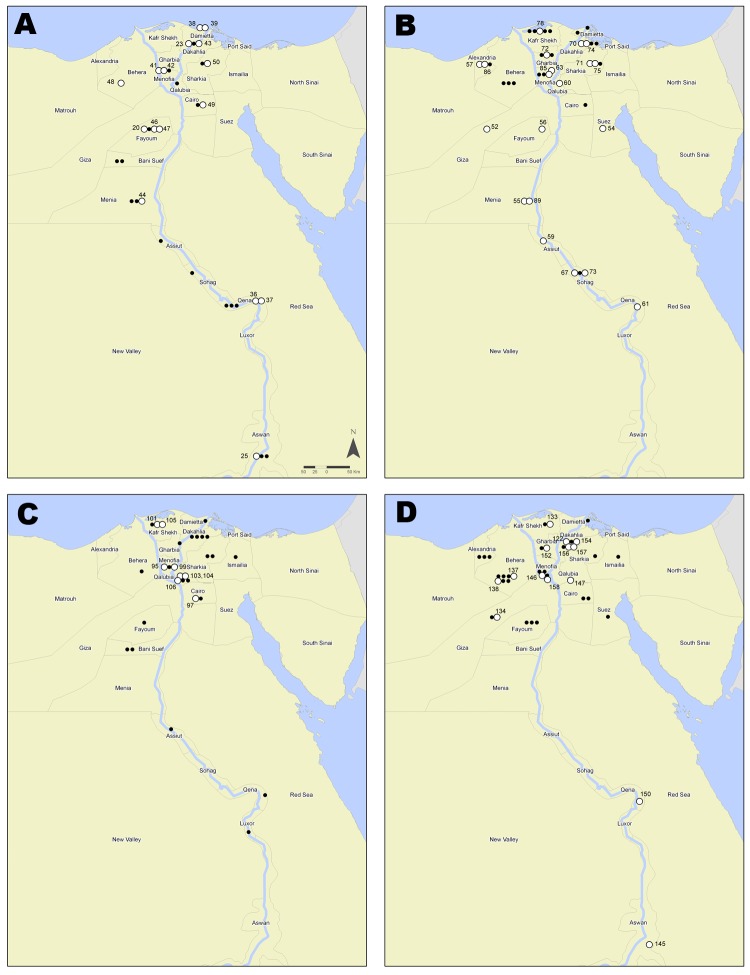
Geographic distribution of humans with highly pathogenic avian influenza A(H5N1) virus infection yielding clade 2.2.1 isolates, Egypt, 2007–2011. Each case is shown within the governorate that reported the case; however, locations within governorate territories are arbitrary and do not represent exact coordinates. White circles indicate the 59 confirmed cases from this study with fully sequenced viral genomes; numbers are the corresponding World Health Organization case numbers. Black dots indicate confirmed cases. Cases include 25 from 2007 and 8 from 2008 (A); 39 from 2009 (B); 29 from 2010 (C); and 39 from 2011 (D).

Additional statistical support for tree topology was assessed by performing Bayesian analyses, using the same datasets as described above and below. Bayesian posterior probabilities were estimated under a Markov Chain Monte Carlo method with 50 million generations implemented in BEAST, using a SRD06 substitution model (*12*). Strain A/turkey/Turkey/1/2005 was used to root all trees. We generated phylogenetic trees in a manner similar to that described above (for HA) for each gene segment from viruses that had full genomes available; representative viruses are shown in Technical Appendix 2 Figure 1. Trees for each gene with all sequences analyzed for this study are shown in Technical Appendix 2 Figure 2.

Viral proteome characterization was conducted after full-length open reading frame nucleotide sequences were aligned using MUSCLE and trimmed to begin at the ATG start codon. For the HA protein sequence analysis, amino acid numbering was based on the mature HA protein sequence after removal of the signal peptide.

### Antigenic Analysis

Antigenic characterization was performed by using a panel of ferret antisera in hemagglutination inhibition (HI) tests with turkey erythrocytes, as described (*13*) (Technical Appendix 2 Table). We used turkey erythrocytes to better resolve antigenic distances between variants identified in this study. All antisera were treated with the receptor-destroying enzyme neuraminidase (NA) from *Vibrio cholerae* (RDE [receptor destroying enzyme]; Denka Seiken Co., Ltd., Tokyo, Japan), according to the manufacturer’s recommendation, and pre-adsorbed with turkey erythrocytes.

### Antiviral Susceptibility Testing

To determine the antiviral drug concentration required to inhibit 50% of the NA activity, we conducted a fluorescent NA inhibition assay as described (*14*). The assays were conducted under biosafety level 3–enhanced containment at CDC.

## Results

### Epidemiology of Subtype H5N1 Infections

Most of the 158 persons in Egypt infected with subtype H5N1 virus during 2007–2011 were residents of the Nile Delta region, north of Cairo (Figure 2). The cases were geographically distributed in 23 of the 27 governorates in Egypt. As determined by Kandeel et al. (*2*), women >15 years of age were at greatest risk for infection and death; 36% of infections and 69% of deaths were in women in this age group (*2*). Risk ratios (RRs) for death included female sex (RR 2.16, p = 0.002), age >15 years (RR 10.26, p<0.0001), and receiving the first dose of oseltamivir >2 days after illness onset (RR 4.15, p<0.0001).

### Genomic Phylogeny of Subtype H5N1 Virus Isolates from Humans

We sequenced the complete genomes of 59 subtype H5N1 virus isolates and 13 HA genes sequenced directly from clinical specimens that were obtained from a total of 72 persons in Egypt during 2007–2011. The phylogenetic trees of the HA gene sequences were annotated according to classification criteria established by the World Health Organization (WHO), the World Organisation for Animal Health, and the Food and Agriculture Organization, and clades were further subdivided as reported (*6*,*8*,*13*).

The phylogenetic tree of the HA gene showed that the 72 genes from the infected persons (Figure 1, coded dots) have evolved into 4 groups that diverged from the ancestral A/turkey/Turkey/2005-like genes. Three groups identified within clade 2.2.1 were supported by bootstrap values >89 and were designated 2.2.1-B, 2.2.1-C, and 2.2.1-D. Clade 2.2.1.1 formed the fourth group, supported by a bootstrap value of 100. All viruses collected in 2011 (n = 23) were clustered within 2.2.1-C, and 22 of the 23 formed a monophyletic group, supported by a bootstrap value of 94, with an average difference of 26 nt. The rest of the group C viruses, including the Aswan isolate A/Egypt/N0423/2011, originated from a distinct lineage but shared ancestry with the A/Egypt/3072/2010 vaccine candidate virus (Figures 1, 2). Viruses from clade 2.2.1.1 included only 1 isolate from humans, whereas subgroup C of clade 2.2.1 comprised 56 human isolates. The determinants of this difference have not been established.

To detect evidence of genetic reassortment among subtype H5N1 viruses from poultry and zoonotic infections, we compared the topologies of phylogenetic trees from the complete genomes of 90 viruses from Egypt (59 sequenced for this analysis and 31 available in GenBank). The topologies of HA and PB2 trees were identical, indicating an absence of reassortment (Technical Appendix 2 Figure 1). The other 6 trees indicated that these genes either co-evolved with HA (identical tree topology with high bootstrap support) or had insufficient divergence to support unambiguous evolutionary relationship determinations (low bootstrap support). The exception was the nonstructural (NS) gene of a subgroup C virus, A/Egypt/9174-NAMRU3/2009, which formed a monophyletic group (bootstrap value 95) with several subgroup B viruses isolated in the same year, indicating a possible reassortment of NS gene between these co-circulating subgroups in 2009.

We also assessed the possible transboundary movement of subtype H5N1 viruses by analyzing the phylogenetic relationships between viruses from Egypt (2007–2011) and other countries. All the genes from viruses from Egypt formed single monophyletic groups, which included isolates exclusively from Egypt (Technical Appendix 2 Figures 1, 3), and were distinct from other closely related genotype Z viruses, including clade 2.2 viruses previously circulating in other regions of Africa, the Middle East, and Europe (*15*,*16*). These results indicated that no additional subtype H5N1 viruses were imported into Egypt after their introduction in 2005/2006. The data also indicate that subtype H5N1 viruses have not reassorted with low pathogenic avian influenza (LPAI) viruses since their initial introduction (Technical Appendix 2 Figure 1).

### Characterization of the Viral Proteome of 2011 Human Isolates

The 10 major viral proteins encoded by the subtype H5N1 genomes were screened to identify variations at sites previously associated with functional traits of public health consequence (Table) (*17*–*24*). The multibasic cleavage site common to clade 2.2.1 HA proteins (^321^PQGEKRRRKKR↓G^333^) remained unchanged in viruses collected in 2011 from infected humans. As previously noted, viruses belonging to a subset of the clade 2.2.1.1 group collected during 2009–2011 lacked a single basic residue in the HA cleavage site that was present in clade 2.2.1 and 2.2.1.1 cleavage motifs (*8*).

**Table:** Signature amino acids of avian influenza A(H5N1) viruses infecting humans in Egypt since 2007*

Protein, amino acid position	Virus group†						Functional relevance (reference#)
	2.2.1-C	9174‡	2009 variants§	2007–2008 variants¶	A, B, and D	2.2.1.1	
PB2							
80	**R**	**R**	**R**	**R**	K	K	NP binding site (*17**,**18*)
129	**N**	**N**	**N**	**N**	T	T	
292	**M**	**M**	I	I	I	I	No known function
PB1							
384	**S**	**S**	**S**	L	L	L	cRNA binding (*19*)
PB1-F2							
40	**G**	**G**	D/G	D	D	D	No known function
PA							
400	**T**	**T**	S	S	S	S	No known function
615	**R**	**R**	K/R	K	K	K	615R mammalian host adaptation (*20*)
HA							
43	**N**	**N**	**N**	**N**	D	D	Antigenic site C (*21*)
120	**N**	**N/D**	**N**	**N**	S	S	No known function
129	**Del**	**Del**	**Del**	**Del**	S	L/S	Near or adjacent to the receptor binding site 130-loop (*21*)
151	**T**	**T**	**T**	**T**	I	I	Antigenic site B and receptor binding (*21*)
NA							
224	**M**	**M**	**M**	L	L	L	No known function
450	**G**	**G**	**G**	S	S	S	No known function
M1							
95	**K**	**K**	**K**	R	R	R	No known function
168	**T**	**T**	I	I	I	I	RNP binding site (*22*)
207	**S**	**S**	N	N	N	N	
M2							
51	**F**	**F**	C	C	C	C	No known function
NS1							
48	**S**	N	**S**	N	N	N	RNA binding site (*23*)
198	**V**	**V**	I/V	I	I	I/V	Effector domain: inhibition of maturation and exportation of host antiviral mRNAs (*23*)
229	**E**	**E**	K	K	K	K	PDZ ligand motif of HPAI equals ESEV (*23*,*24*)

*Hemagglutinin (HA) amino acid numbering was based on the mature HA protein sequence after removal of the signal peptide. All other numbering was relative to the full-length open reading frame of A/goose/Guangdong/1/1996 protein sequences. PB2, polymerase basic 2 gene; NP, nucleoprotein; PB1, PB 1 gene; PB1-F2, alternate open reading frame near the 5′ end of the PB1 gene; PA, polymerase acidic gene; Del, deletion; NA, neuraminidase; M, matrix gene; RNP, ribonucleoprotein; NS, nonstructural gene; PDZ, postsynaptic density protein, *Drosophila* disk large tumor suppressor, and zonula occludens-1 protein; HPAI, highly pathogenic avian influenza; ESEV, H5N1 PDZ-binding motif amino acid consensus sequence. †**Boldface** indicates unique amino acid differences found in the various positions along the different genes of 2.2.1-C viruses. ‡Indicates intraclade reassortant virus A/Egypt/9174-NAMRU3/2009. §A/Egypt/0606-NAMRU3/2009, A/Egypt/2752-NAMRU3/2009, A/Egypt/3450-NAMRU3/2009. ¶Early 2.2.1-C viruses from 2007–2008: A/Egypt/394-NAMRU3/2007, A/Egypt/2546-NAMRU3/2008, A/Egypt/2289-NAMRU3/2008. #References describing functional significance of mutations.

The HA proteins of the clade 2.2.1 viruses from 2011 also lacked glycosylation at position 154, as observed in older H5N1 viruses from Egypt. However, A/Egypt/N10621/2011 and A/Egypt/N6658/2011 acquired an additional glycosylation site at Asn 119 and 215, respectively, in the globular head of HA. Two signature amino acid changes (a deletion of Ser 129 in the HA gene, and an Ile 151 Thr substitution) were observed in HA proteins of clade 2.2.1 group C viruses collected during 2007–2011; both changes may enhance binding to α-2,6–linked receptors (*8*,*13*,*25*). Additional amino acid comparisons revealed that the HA of the virus from the Aswan region of Egypt lacked the amino acid signatures shared among the viruses collected in 2011 from northern Egypt. However, the HA gene from this virus featured amino acid signatures shared among older group 2.2.1-C viruses isolated in southern and northern Egypt (Table).

Additional polymorphisms of potential relevance in clade 2.2.1-C viruses included the K615R mutation in the polymerase acidic (PA) gene, previously reported to be associated with mammalian host adaptation (*20*). Several additional polymorphisms were also found within the known functional domains of the internal genes, such as those involved in RNA and/or nucleoprotein binding (*17*–*24*) (Table). Two of the isolates (A/Egypt/N6828/2011 and A/Egypt/N7562/2011) possessed a stop codon at position 25 in the polymerase basic 1 (PB1)–F2 coding region (alternate open reading frame near the 5′ end of the PB1 gene), causing a truncated protein. One of the poultry viruses also possessed a premature stop codon at position 9 in PB1-F2. A single poultry virus, A/chicken/Egypt/083/2008, had only a 22-aa PA-X protein, and all of the human and other poultry viruses had PA-X proteins comprising the full 61-aa sequence. No distinct amino acid signatures were found in the nucleoprotein or NS2 proteins. All of the clade 2.2.1-C viruses and the other clade 2.2.1 and 2.2.1.1 viruses retained PB2 627K and NS1 92E, two mutations imparting host-specific virulence phenotypes in subtype H5N1 viruses, that are also conserved in nearly all clade 2.2-origin viruses isolated from humans and birds (*26*,*27*).

### Antigenic Characterization of Recent Subtype H5N1 Isolates from Humans

To assess the extent of antigenic drift among recent human subtype H5N1 virus isolates relative to WHO-recommended candidate vaccine viruses, we performed hemagglutinin inhibition (HI) tests using ferret antisera raised against a panel of viruses, including A/Egypt/N03072/2010 (the clade 2.2.1 vaccine candidate recommended by WHO) (*28*) and isolates from previous years. Antiserum generated against A/Egypt/N03072/2010 revealed HI titers to representative subtype H5N1 isolates from 2011 that differed <4-fold, compared with the homologous titer of the candidate vaccine virus (Technical Appendix 2 Table). Compared with A/Egypt/N03072/2010, most of the analyzed viruses from humans infected in 2011 had 6–8 aa changes in the HA1 protein (Technical Appendix 3). Relative to the vaccine strain, these viruses shared 3 conserved amino acid differences at positions previously shown to be involved in antibody recognition (D154N and N155D) or receptor binding (V134A). Relative to the homologous titer, HI titers of antiserum to A/Egypt/N03072/2010 were reduced by 8-fold with A/Egypt/N6828/2011 and A/Egypt/N7562/2011 (Technical Appendix 2 Table). A/Egypt/N6828/2011 possessed a change at putative antigenic site A (A127T) relative to other group C viruses, but there were no other additional amino acid substitutions at known functional sites to support this finding (Technical Appendix 3).

The HI titers of antisera to earlier clade 2.2.1 viruses (groups A, B, and D) with group C viruses from 2011 were reduced >8-fold relative to the homologous values, indicating substantial antigenic change in recent years. These earlier viruses had many other additional HA1 substitutions in comparison to the group C viruses, including several in or near to antigenic sites B and C (Technical Appendix 3). In addition, only the group C viruses (including the vaccine virus) shared a deletion of amino acid residue 129, which is near or adjacent to the 130-loop of the HA receptor-binding site (*25*). The large number of changes at immunodominant sites between the clade 2.2.1.1 viruses and group C viruses, together with a lack of antigenic crossreactivity in 2-way tests, indicate disparate evolutionary paths of these lineages. In agreement with previous findings (*13*), we showed that sera generated against the clade 2.2.1.1 viruses (e.g., A/chicken/Egypt/9403-NAMRU3/2007 and the A/Egypt/3300-NAMRU3/2008 vaccine candidate) did not crossreact with the group C viruses.

### Antiviral Drug Susceptibility

Analyses of putative molecular markers of resistance to antiviral drugs (adamantanes and NA inhibitors) identified few variations of concern for public health. A/Egypt/4396-NAMRU3/2009 had an Ile-to-Thr mutation at position 223 (I223T) in the NA active site; however, this change did not affect susceptibility of the virus to oseltamivir (50% inhibitory concentration 5.23 nM). A/Egypt/0605-NAMRU3/2009 had a mutation of I117M in the NA, and compared with the sensitive control virus, which lacked this mutation, A/Egypt/0605-NAMRU3/2009 had a 6-fold reduced susceptibility to oseltamivir (16.46 nM vs. 2.68 nM). Four viruses had the V27A mutation in the M2 protein (A/Egypt/ 2289/NAMRU3/2008, A/Egypt/2546/NAMRU3/2008, A/Egypt/N6774/2011, and A/Egypt/N7724/2011), and 1 virus had the S31N mutation (A/Egypt/3300/NAMRU3/2008). These viruses are predicted to be resistant to M2 ion-channel blocking drugs.

## Discussion

Since 2009, Egypt has reported a higher number of HPAI (H5N1) virus infections in humans than any other country. At the end of 2011, Egypt had reported 39 (63%) of the total 62 human cases in the world for that year, placing Egypt second only to Indonesia in the number of reported human infections since 2003 (*7*). Exposure to sick or dead poultry has been reported as the likely source of infection for nearly all human cases in Egypt (*29*). Most of those exposures were described as occurring in backyard poultry settings (although infection in industrial/commercial settings could not be ruled out if someone worked in or visited these settings) (*29*). The contrast between the rising numbers of human infections detected each year in Egypt and the declining case-fatality ratios since 2009 led to speculation about the evolution of new virologic properties influencing the epidemiology of subtype H5N1 virus in Egypt (*2*,*8*,*25*). To investigate possible molecular epidemiologic correlates of these trends, we analyzed the complete genomes of viruses isolated from humans in Egypt during 2007–2011.

Phylogenetic analysis of HA genes indicated that subtype H5N1 viruses from clades 2.2.1 and 2.2.1.1 continued to co-circulate in recent years (*6*). In addition, the HA of clade 2.2.1 viruses was also found to cluster in 1 of 4 distinct monophyletic groups (previously termed groups A–D) (*8*,*13*). Of the human infections during 2009–2011, 95% were caused by viruses from a single phylogenetic group, clade 2.2.1-C, whereas infections detected during 2007–2008 involved viruses from 2.2.1-B and -D.

In contrast with the multiple human infections caused by the clade 2.2.1 viruses, only 1 human infection by a clade 2.2.1.1 virus was detected (Figure 1). The predominance of clade 2.2.1-C viruses among zoonotic infections in humans appears to be associated with the persistent circulation of this group of viruses in backyard or peridomestic poultry (30). However, there remains some evolutionary divergence between 2 discrete HA clusters detected in 2011 that may correlate with the geographic separation between the majority of group C genes that originated from the Nile Delta and those from strain A/Egypt/N0423/2011, which was collected from the Aswan governorate in the south of the country. The paucity of human infections with clade 2.2.1.1 viruses, which circulate predominantly in commercial poultry (*30*), may result from intrinsic viral properties or from husbandry practices that reduce the probability of zoonotic infection. Thus, exposure to subtype H5N1 virus in backyard or peridomestic environments, rather than commercial settings, appears to be correlated with greater risk for zoonotic H5N1 infections.

The genetic variation that was observed among the HA genes of clade 2.2.1 viruses correlated with an increased complexity in the antigenic characteristics of the viruses currently circulating in Egypt. Although all clade 2.2.1 viruses remain crossreactive to sera produced against reference viruses from the same clade, HI assay results indicated that continued variation among A, B, C, and D viruses has resulted in reduced titers of recent 2.2.1-C viruses against antisera to older clade 2.2.1 viruses. It should be noted, that the recent 2011 isolates were antigenically closely related to the proposed WHO candidate vaccine virus, A/Egypt/N03072/2010, indicating a good antigenic match between currently circulating strains and the proposed vaccine.

Although the HA and NA genes play a major role in the transmission of HPAI (H5N1) virus, the internal genes can also modulate pathogenicity and transmissibility of the virus (*17*–*27*). To further investigate the evolution of the internal genes of subtype H5N1 viruses from humans and detect possible reassortment (intraclade or other), we performed a systematic analysis of the phylogenetic relationships among all the genes. There was a notable difference in the topology of the phylogenetic tree of the NS gene compared with other internal genes (supported by bootstrap values >80) in that no distinct 2.2.1-B and 2.2.1-D subgroups were evident for the NS gene tree. All the internal genes that did not appear to co-evolve with their surface genes show the closest relationship to the genes of A/turkey/Turkey/2005, indicative of a lack of strong selective pressure. These findings also confirmed previous reports that subtype H5N1 genes have not been introduced from Asia into Egypt since 2006. The phylogenies of all the genes from the viruses in Egypt lacked evidence of reassortment with other avian influenza genes.

The co-circulation of H9N2 and potentially other LPAI A viruses in Egypt has been reported (*31*); thus, the absence of genetic reassortment between subtype H5N1 and LPAI avian viruses from poultry could be considered unexpected. In contrast to reassortant genotypes that have been identified in other countries, the homogeneous genetic makeup of subtype H5N1 viruses in Egypt may stem from the characteristics of the poultry trade with neighboring countries or from other factors leading to fewer opportunities for co-infections with other avian influenza viruses.

At the protein level, evolution among 2.2.1-C viruses, compared with that among other clade 2.2.1 viruses, has resulted in the fixation of at least 4 substitutions in the HA protein. When found together, 2 of the 4 HA mutations have been implicated as possible host adaptation markers: the deletion of residue 129 and the I151T substitution enabled in vitro binding to α-2,6 sialosides and virulence in mice (*25*). However, these 2 mutations may also mediate antigenic drift (*20*). The K615R substitution in the PA proteins of 2.2.1-C viruses represents another potential marker of host adaptation (*17*). No markers of antiviral drug resistance were found to be conserved in either the NA or M2 of any of the human-derived subtype H5N1 viruses. Epidemiologic investigations of the human cases of subtype H5N1 infection from which the study isolates were derived could not confirm which patients had received oseltamivir or other antiviral treatments. However, previous analyses of subtype H5N1 viruses isolated from poultry in Egypt were found to have M2 and NA proteins with antiviral resistance markers, indicating that these mutations exist to some extent in viruses circulating within the poultry population (*8*).

In summary, our findings indicate that the recent subtype H5N1 viruses isolated from human infections originated from poultry. These viruses evolved from a single genotype introduced into Egypt in 2005/2006; there is no evidence of subsequent reassortment with new subtype H5N1 virus genes introduced into Egypt or resident LPAI viruses. The viruses have been observed in all subtype H5N1 infections in humans since 2009 and belong to a subgroup, termed 2.2.1-C, with unique genetic signatures that may contribute to their persistence in poultry. A rationale for linking the observed amino acid changes to the decline in case-fatality ratios since 2009 could not be identified. Systematic analysis of subtype H5N1 viruses in Egypt is critical to better understand the genetic and phenotypic evolution of subtype H5N1 viruses in Egypt and to inform public health programs to reduce the risk for zoonotic infections and prevent or mitigate a potential pandemic.

Technical Appendix 1 Genetic database accession numbers and World Health Organization case report data for highly pathogenic avian influenza A(H5N1) viruses isolated from humans, Egypt, 2007–2011.

Technical Appendix 2 Phylogenetic trees showing the relationships among highly pathogenic avian influenza A(H5N1) viruses isolated from humans, 90 viral genomes that were analyzed, phylogeny of the complete genomes of subtype H5N1 viruses in Egypt, 2007–2011, and antigenic analysis of avian subtype H5N1 viruses isolated from humans in Egypt during January–September 2011.

Technical Appendix 3 Amino acids of representative highly pathogenic avian influenza A(H5N1) viruses isolated from humans in Egypt compared with those of the World Health Organization–recommended clade 2.2.1 vaccine candidate, A/Egypt/N03072/2010.
