# Supplementary material for: Microevolution of Highly Pathogenic Avian Influenza A(H5N1) Viruses Isolated from Humans, Egypt, 2007–2011
Source: Emerg Infect Dis. 2013 Jan;19(1):43–50. doi: 10.3201/eid1901.121080 (PMC3563221; doi:10.3201/eid1901.121080)
Supplement: Technical Appendix 2 — Phylogenetic trees showing the relationships among highly pathogenic avian influenza A(H5N1) viruses isolated from humans, 90 viral genomes that were analyzed, phylogeny of the complete genomes of subtype H5N1 viruses in Egypt, 2007–2011, and antigenic analysis of avian subtype H5N1 viruses isolated from humans in Egypt during January–September 2011. [file 12-1080-Techapp2-s2.pdf]

Article DOI: http://dx.doi.org/10.3201/eid1901.121080

Microevolution of Highly Pathogenic Avian Influenza A(H5N1) Viruses Isolated from Humans, Egypt, 2007–2011

Technical Appendix 2

Technical Appendix 2 Figure 1 (next page). Phylogenetic relationships among highly pathogenic avian influenza A(H5N1) viruses isolated from humans, Egypt, 2007–2011. Trees were built on the basis of representative viral genomes from the 90 isolates analyzed in this study (Technical Appendix 2 Figure 2). Bootstrap values >79 generated from 1,000 neighbor-joining replicates are shown above branches, and Bayesian posterior probabilities are shown below the branches at relevant nodes. The trees were rooted to the A/turkey/Turkey/1/2005 genes. The viruses are labeled and colored according to the clade 2.2 subgroups of their HA sequences (i.e., B, D, C, and 2.2.1.1); branch colors in all trees are used similarly to those in the HA tree. Red branches denote human viruses. Triangles, variant viruses with clade 2.2.1-C HA lacking unique genetic signatures in the internal genes (as shown in the Table in the main text); underlining, viruses isolated in 2011; dots, isolate from the Aswan governorate; hash mark, NS gene reassortant. HA, hemagglutinin; NA, neuraminidase; PB2, polymerase basic 2 gene; PB1, PB 1 gene; PA, polymerase acidic gene; NP, nucleoprotein; M, matrix gene; NS, nonstructural gene. Scale bar indicates nucleotide substitutions per site.

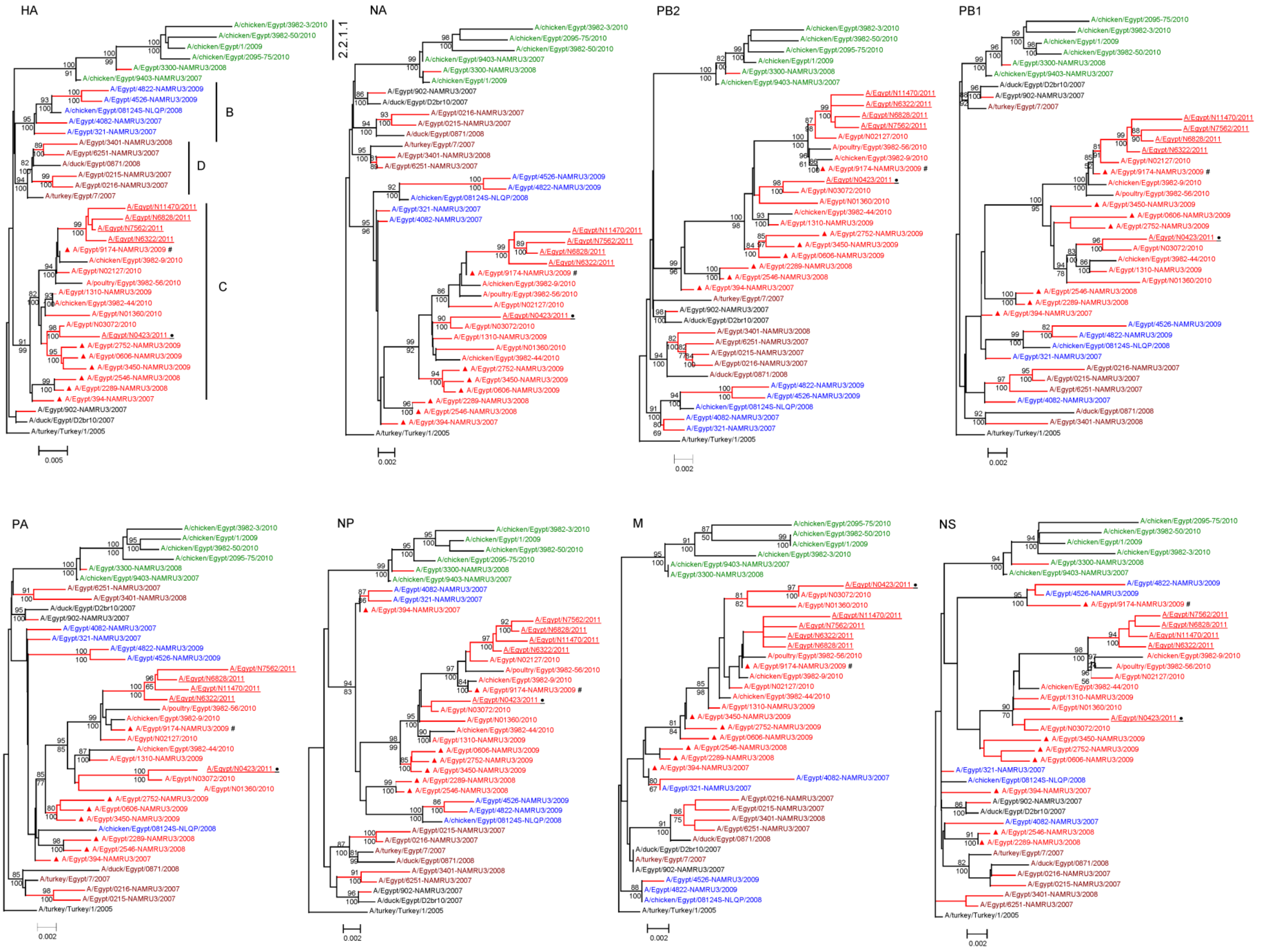

HA
NA
PB2
PB1
PA
NP
M
NS
2.2.1.1
B
D
C
0.005
0.002

Technical Appendix 2 Figure 2 (next page). Phylogenetic trees of 90 viral genomes analyzed in a study of highly pathogenic avian influenza A(H5N1) viruses isolated from humans, Egypt, 2007–2011. The sequences of the 8 viral genes were aligned and trees were constructed as described for Technical Appendix 2 Figure 1. Annotation conventions and abbreviated terms are similar to those used for Technical Appendix 2 Figure 1. Sequences labeled Representative sequences, labeled as REP, were included in Technical Appendix 2 Figure 1. Scale bars indicate nucleotide substitutions per site.

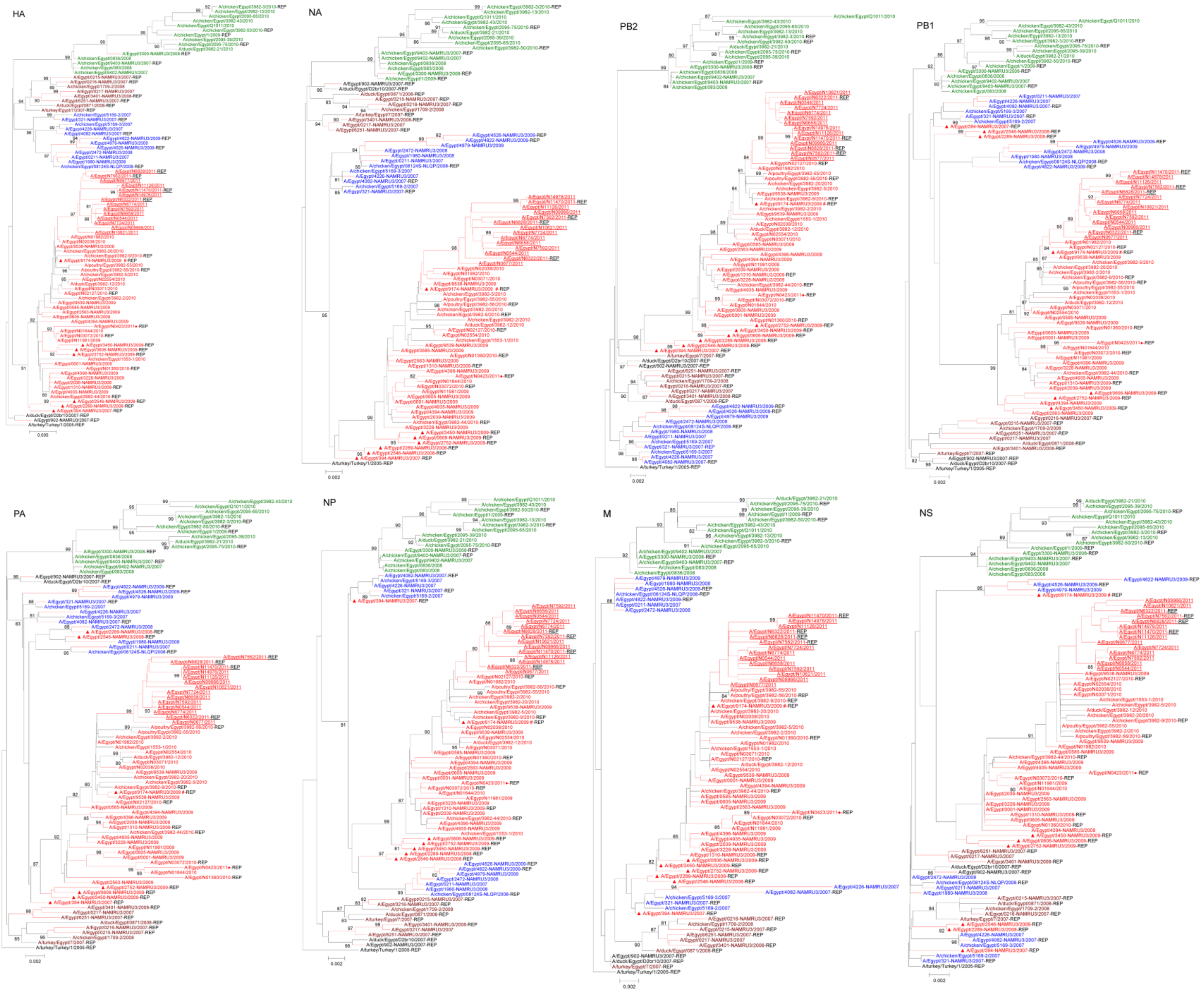
HA
0.005
NA
0.002
PB2
0.002
PB1
0.002
PA
0.002
NP
0.002
M
0.002
NS
0.002

Technical Appendix 2 Figure 3. Phylogeny of the complete genomes of highly pathogenic avian influenza A(H5N1) viruses in Egypt, 2007–2011. The sequences of the internal genes belonging to the 90 viruses analyzed in this study were aligned with representative viruses from other countries from different lineages, and trees were built in MEGA5 (www.megasoftware.net), using the neighbor-joining method based on a maximum composite likelihood model. Bootstrap values >79 generated from 1,000 neighbor-joining replicates are shown on the branches. PB2, polymerase basic 2 gene; PB1, PB 1 gene; PA, polymerase acidic gene; NP, nucleoprotein; M, matrix gene; NS, nonstructural gene. Scale bars indicate nucleotide substitutions per site.

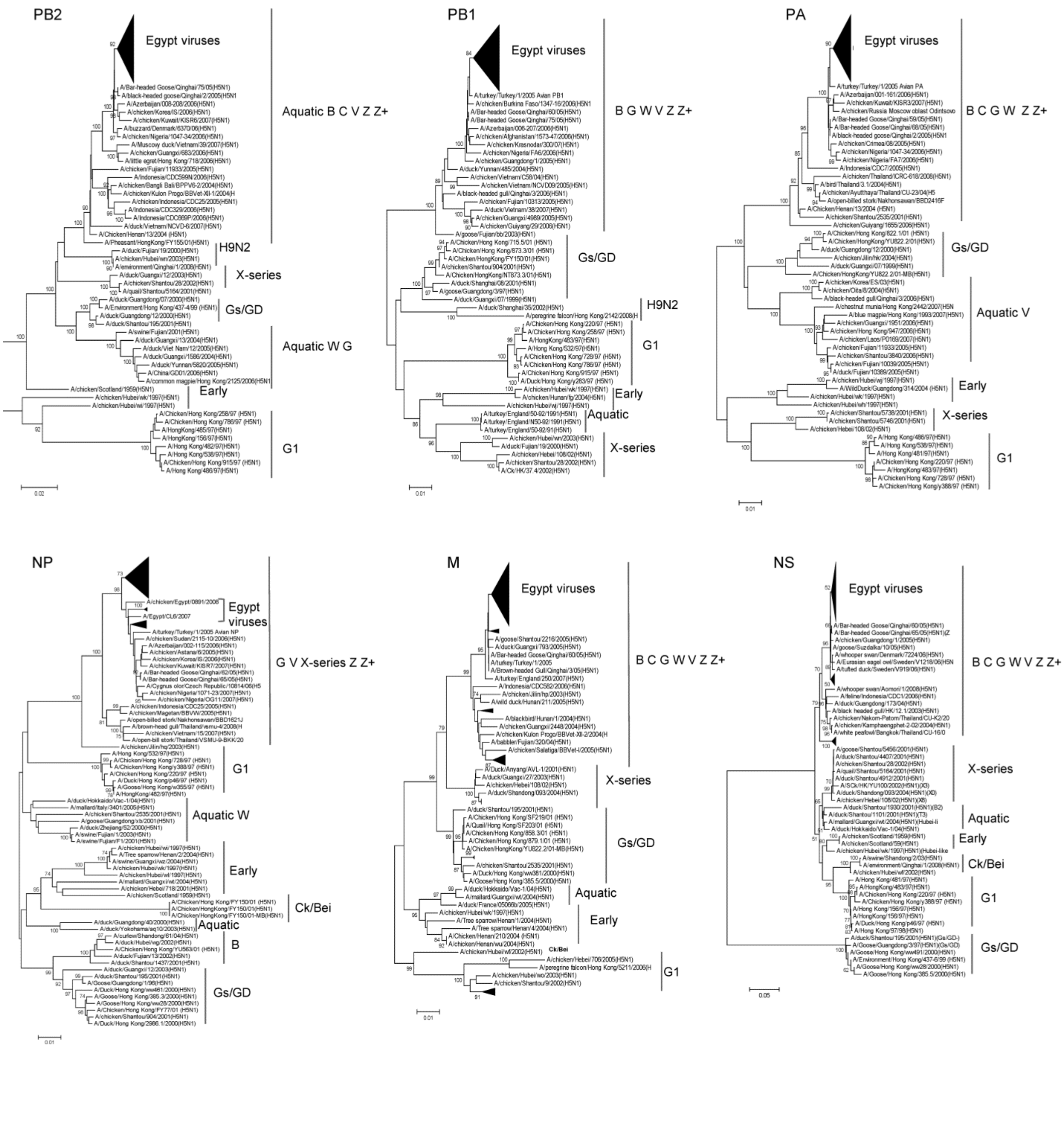

Table . Antigenic analysis of avian influenza A(H5N1) viruses isolated from humans in Egypt during January–September 2011*

		Reference ferret antisera clade, reference antigen												
				2.2.1, group										
Antigen	Clade/ Egypt group	1, VN/04	2.2, ws/MG	2.2.1, tk/TK	A, eg/EG	B, EG/ 321	C, EG/ 9539	C, EG/ 3072	C, EG/ 2289	C, EG/ 2546	D, EG/ 0215	2.2.1.1, ck/ EG	2.2.1.1, EG/ 3300	2.3.4, ANH/1
Reference antigens														
A/Vietnam/1203/2004	1	**160**	10	10	5	10	5	10	5	10	5	5	5	5
A/ws/MG/244/2005	2.2	5	**1280**	640	320	160	5	1280	640	160	80	20	5	5
A/turkey/Turkey/1/2005	2.2.1	5	1280	**2560**	320	160	80	1280	640	5120	160	10	5	5
A/eg/Egypt/1162-N3/06	2.2.1-A	5	2560	1280	**640**	320	40	1280	640	160	160	20	5	5
A/Egypt/321-N3/2007	2.2.1-B	5	640	320	160	**160**	20	640	160	40	20	5	5	5
A/Egypt/9539-N3/2009	2.2.1-C	5	640	640	160	320	**320**	2560	2560	640	160	5	5	10
A/Egypt/N03072/2010	2.2.1-C	5	1280	1280	320	320	320	**2560**	2560	640	160	10	5	10
A/Egypt/2289-N3/2008	2.2.1-C	5	2560	2560	640	640	1280	5120	**5120**	2560	1280	640	5	320
A/Egypt/2546-N3/2008	2.2.1-C	5	2560	2560	320	320	640	5120	5120	**1280**	640	320	5	320
A/Egypt/0215-N3/2007	2.2.1-D	5	1280	640	320	160	40	640	320	160	**320**	10	10	5
A/ck/Egypt/9403N3/07	2.2.1.1	5	5	5	5	80	5	5	5	5	5	**640**	5	5
A/Egypt/3300-N3/2008	2.2.1.1	5	5	5	5	10	5	5	80	10	10	320	**2560**	5
A/Anhui/1/2005	2.3.4	5	40	40	5	20	5	10	10	20	10	10	5	**640**
Test antigens														
A/Egypt/N0544/2011	2.2.1-C	5	160	320	80	40	80	640	640	160	5	5	5	5
A/Egypt/N6322/2011	2.2.1-C	5	320	320	40	40	80	640	640	160	20	5	5	5
A/Egypt/N6658/2011	2.2.1-C	5	640	1280	160	80	320	1280	1280	320	40	5	5	5
A/Egypt/N0677/2011	2.2.1-C	5	320	320	10	40	80	640	640	160	10	5	5	5
A/Egypt/N6774/2011	2.2.1-C	5	320	320	5	40	160	640	640	160	10	5	5	5
A/Egypt/N6828/2011	2.2.1-C	5	160	160	10	20	10	320	320	80	10	5	5	5
A/Egypt/N7562/2011	2.2.1-C	5	80	160	20	20	40	320	320	80	20	5	5	5
A/Egypt/N7592/2011	2.2.1-C	5	640	640	80	80	160	1280	1280	160	10	5	5	5
A/Egypt/N7724/2011	2.2.1-C	5	640	1280	320	160	640	1280	1280	640	80	5	5	80

*Bold indicates homologous titer.
